# Flow Cytometric Assessment of Erythrocyte Shape through Analysis of FSC Histograms: Use of Kurtosis and Implications for Longitudinal Evaluation

**DOI:** 10.1371/journal.pone.0059862

**Published:** 2013-03-21

**Authors:** Christoph Ahlgrim, Torben Pottgiesser, Thomas Sander, Yorck Olaf Schumacher, Manfred W. Baumstark

**Affiliations:** 1 Department of Rehabilitative and Preventive Sports Medicine, Freiburg University Medical Center, Freiburg, Germany; 2 Aspetar Orthopedic and Sports Medicine Hospital, Doha, Qatar; University of Sao Paulo - USP, Brazil

## Abstract

Sphericity of erythrocytes can be estimated from analysis of FSC signal distribution in flow cytometry. Previously, Pearson’s coefficient of dissymmetry (PCD) and spherical index (SphI) were applied to determine erythrocyte sphericity from the FSC histogram. The aim of the present study is to illustrate the application of kurtosis as an indicator of erythrocyte sphericity in flow cytometry in a broad range of FSC distributions. Moreover, the possibility of longitudinal evaluation of erythrocyte sphericity is studied. Change of erythrocyte sphericity of 10 healthy subjects was induced by variation of buffer osmolarity to validate applicability of sphericity measures. Agreement between the sphericity indicators was then studied in samples from 20 healthy donors taken at three time points, which were processed through density gradient centrifugation and incubated with FITC-labelled antibodies to induce a broad variation of erythrocyte form (1086 samples). SphI, PCD and kurtosis of FSC distribution were calculated. Correlation of the respective measures, standard error of measurement (SEM) and r ratio (intra- to interindividual variance) were determined to illustrate agreement between the sphericity indicators. In the first study part, all sphericity indicators illustrated change of erythrocyte shape as induced by osmolarity variation. In the second part, correlation between kurtosis and SphI was −0.97 and correlation between kurtosis and PCD was 0.58 (p<0.05). In isotype control samples, correlation between kurtosis and SphI was −0.98 and correlation between kurtosis and PCD was 0.48 (p<0.05). In these samples, mean kurtosis was −0.80 (SEM 0.03), mean SphI was 2.19 (SEM 0.04) and mean PCD was −0.31 (SEM 0.02). r ratios of all measures of sphericity were <0.6. Our results show that kurtosis is closely correlated with SphI in a broad range of erythrocyte FSC distributions. Moreover, all measures of sphericity feature r ratios <0.6, highlighting that erythrocyte sphericity appears as a feasible parameter for individual longitudinal data monitoring.

## Introduction

Flow cytometry has been used to assess size and granularity of particles for more than 50 years. The forward scatter (FSC) signal returns the size of the different particles in the analysis in which, usually, a higher FSC signal indicates a higher cellular volume [1]. As a unique feature of erythrocytes due to their biconcave shape, forward scattering of erythrocytes in flow cytometry may show a bimodal distribution of FSC signals [2]. Hence, changes in erythrocyte morphology towards a more spherical shape, e.g. through osmotic properties of their environment, will cause a FSC distribution approaching rather a monomodal form [3]–[5]. The seminal findings of this specific feature of erythrocytes were made more than 30 years ago.

Recently, Piagnerelli and co-workers revived the knowledge about bimodal distribution of erythrocyte’s FSC signal and provided an application with clinical relevance [4], [6]. In brief, they showed that different disease states (e.g. sepsis, chronic renal insufficiency, diabetes) lead to deformation of erythrocytes towards a more spherical shape [4]. They were able to clearly distinguish erythrocytes taken form patients featuring these diseases through the analysis of the FSC distribution and proposed their method as a rapid test for erythrocyte morphology. In their work, Piagnerelli et al. used Pearson’s second skewness coefficient of dissymmetry (PCD) [7] to measure skewness of FSC histogram and introduced a new measure determined as the “spherical index” (SphI), a ratio practically aiming to describe the distance between two peaks of a bimodal distribution.

Kurtosis, like skewness introduced by Karl Pearson in the beginning of the 20^th^ century [8], is a distinct parameter to describe the steepness of distributions [9]. Kurtosis is mathematically referred to as β_2._ By definition, kurtosis (expressed as β_2_) of the normal distribution is 3 [9]. To enable comparison of distributions with the normal distribution, kurtosis is often expressed as “excess kurtosis” (β_2_-3), as excess kurtosis for the monomodal normal distribution is 0. In practical application, kurtosis might constitute a sensitive parameter to distinct bimodal from monomodal distribution [10] as kurtosis declines when approaching bimodality. For the “most bimodal” of all distributions, the symmetric two-point distribution, β_2_-3 is −2 [10].

**Figure 1:**
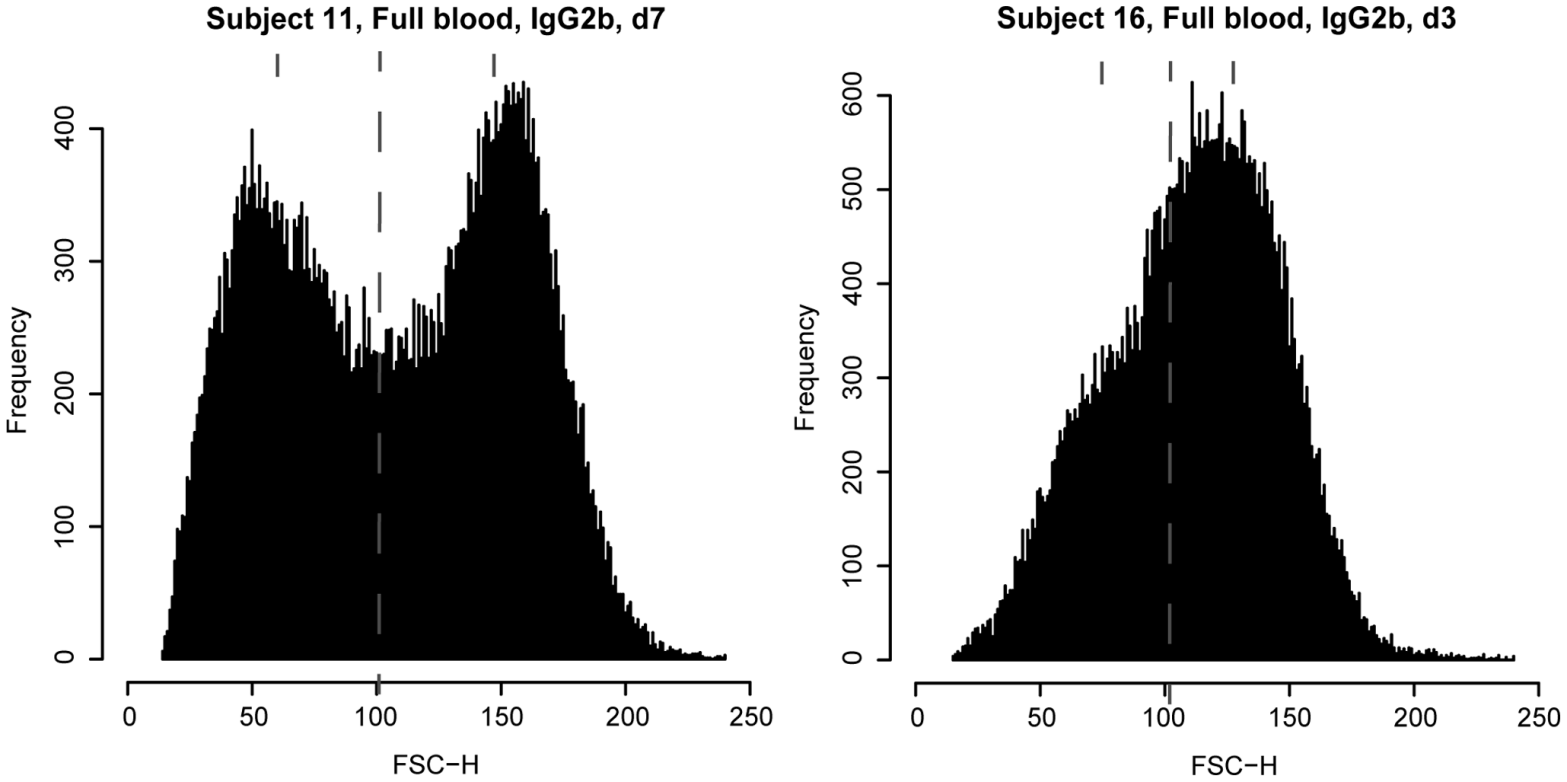
Selected FSC histograms illustrating bimodal and monomodal distributions. Selected FSC histograms illustrating bimodal and monomodal distributions. x-axis displays the channels used for analysis, y-axis gives the number of events for each channel. Dashed lines indicate channel 101 chosen for calculation of SphI. The median value for the respective part of the distribution is marked by short lines. For subject 11 (Full, IgG2b, d7), kurtosis = −1.21, PCD = −0.15, SphI = 2.47 (median 1 = 148, median 2 = 60). For subject 16 (Full, IgG2b, d3), kurtosis = −0.36, PCD = −0.27, SphI = 1.70 (median 1 = 129, median 2 = 76).

In contrast to skewness, kurtosis can distinguish even symmetric bimodal distributions from normal distribution. In further contrast to SphI, kurtosis can be calculated from the original FSC histogram and does not require separating the two parts of the bimodal distribution. Therefore kurtosis might be the more favourable parameter to analyse erythrocyte sphericity using the FSC signal. Figure 1 exemplifies a monomodal and a bimodal FSC distribution and the respective values for kurtosis, PCD and SphI.

The present study aims at comparing the erythrocyte shape indicators PCD and SphI with kurtosis in a large set of erythrocyte samples featuring various types of FSC signal distribution caused by pre-analytical processing and different flow velocities in the flow cytometer. Longitudinal data analysis will, for the first time, provide data on reliability and intra-individual variability of the respective measures.

## Materials and Methods

All subjects participating in this study provided written informed consent to participate in this study, which was approved by the University Hospital ethics committee.

### First Study Part

In the first study part, erythrocyte shape was directly varied in a predictable way through incubation in buffer medium of different osmolarities. Blood samples of 10 healthy donors were obtained in 2.7 ml K+EDTA coated tube (BD Plastipak, Heidelberg, Germany) and washed twice in PBS/2 mM EDTA. Erythrocytes were then resuspended in PBS/2 mM EDTA buffer. Percentage of PBS in this medium was adjusted to achieve buffer osmolarities of 259 mosmol/kg, 285 mosmol/kg, 300 mosmol/kg, 315 mosmol/kg and 335 mosmol/kg. Samples were incubated for 40 minutes to allow the cell shape to adjust to the osmolarity of the surrounding medium. In total 50 samples were analysed.

### Second Study Part

To apply kurtosis in a broad range of FSC signals aimed at studying samples which underwent pre-analytical processing through density separation and antibody labelling. Samples were obtained from an anti-doping research project conducted in our institute. The key objective of this project was to evaluate erythrocyte properties after blood transfusion for anti-doping research in a controlled trial. Key outcomes of the main research project will be reported elsewhere. An experimental group (EXP, n = 10) was transfused with one unit of autologous blood (approximately 280 mL erythrocyte concentrate) 49 days after initial blood donation when full hematologic recovery may be assumed [11]. Blood samples were collected from an antecubital vein once before and twice after re-transfusion of the unit of blood as well as in 10 control subjects (CONT) at the respective time points without performing blood donation and transfusion. Collection and further processing of the blood samples as described below led to a broad range of FSC distributions which could longitudinally analysed:

In total, 60 blood samples were collected into a 2.7 ml K+EDTA coated tube (BD Plastipak, Heidelberg, Germany) at three different time points (one day previous to re-transfusion (d-1) and three (d3) and seven days (d7) post re-transfusion). All samples were washed twice with PBS/2 mM EDTA buffer. The samples, except for “Full” (which were analyzed on the flow cytometer without further treatment, (see below)) were further processed through a 4-layer, isoosmotic, discontinuous density gradient (stock solution 10 v% of Sorbitol 40% mixed with 90 v% of Percoll (GE Healthcare, Freiburg, Germany), further diluted with PBS/2 mM EDTA buffer to solutions of 60 v%, 68 v%, 80 v% and 90 v%. Respective densities were 1.070, 1.099, 1.106 and 1.116 g/ml. Blood was transferred to the gradient medium. Fractions “Low”/”Mean”/”High” were taken from the cell bands appearing after centrifugation for 20 min×1080 g. Blood was then washed from the density gradient medium. Detailed results of density gradient processing will be reported elsewhere.

All samples were subsequently incubated with FITC-labelled antibodies against CD44, CD47, CD58, CD59 and CD71 and three corresponding FITC-IgG-antibodies (IgG1, IgG2a, IgG2b), which were used as an isotype control (all antibodies: BD Pharmigen FITC mouse IgG antibody class, BD Biosciences, Heidelberg, Germany). In total 1086 samples of erythrocyte/antibody solution were created. All samples were always kept in isoosomolarity.

Samples of both study parts were analysed on a BD FacsCalibur flow cytometer (Becton, Dickinson, Heidelberg, Germany) using the primary 488 nm laser beam, the FSC channel was set to linear. Sheath fluid (BD Facsflow, Erembodegem, Belgium) was isoosmotic. For “Full”, “High” and “Mean”, 50.000 events were recorded at “low” flow rate (12 µl/min). For “Low” 10.000 events were recorded at “high” flow rate (60 µl/min).

### FACS Data Analysis

FACS data was analysed using the flowcore package (Release 2.10, Bioconductor, open source) available for the R statistics software (Version 2.15.1, THE R Foundation for Statistical Computing, GNU General Public License). Kurtosis, the fourth moment of the FSC distribution, was calculated as outlined in detail by Joanes and Gill [12] using the e1071 package (Version 1.6) [13], with G_2_ preset in R (kurtosis expressed as β_2_-3). PCD was calculated as explained by Yule and Kendall [7]. SphI was calculated as indicated by Piagnerelli et al. [4].

### General Statistics

Friedman-test with adjusted pairwise comparisons was used to determine effect of osmolarity on erythrocyte shape in the first study part. Correlation of samples was analysed using Spearman’s rank-based correlation coefficient (ρ). Calculations were performed with IBM SPSS 20.0 (IBM Deutschland, Ehningen, Germany). Intra- versus inter-subject variability of the respective measures is expressed as ratio r, which was introduced by Harris et al. [14]. When r is small, the variance of the intra-individual distribution of measurements is smaller than the overall variance of samples taken from a population. For r<0.6 a population based distribution will include a larger-than-expected proportion of an individual’s values. Variance analysis for calculation of r was performed in R using a linear mixed-effects model fitted by maximum likelihood [15]. Standard error of measurement (SEM) was calculated as intra-individual-variance/√(n). P<0.05 was accepted for statistical significance.

## Results

### First Study Part

**Figure 2:**
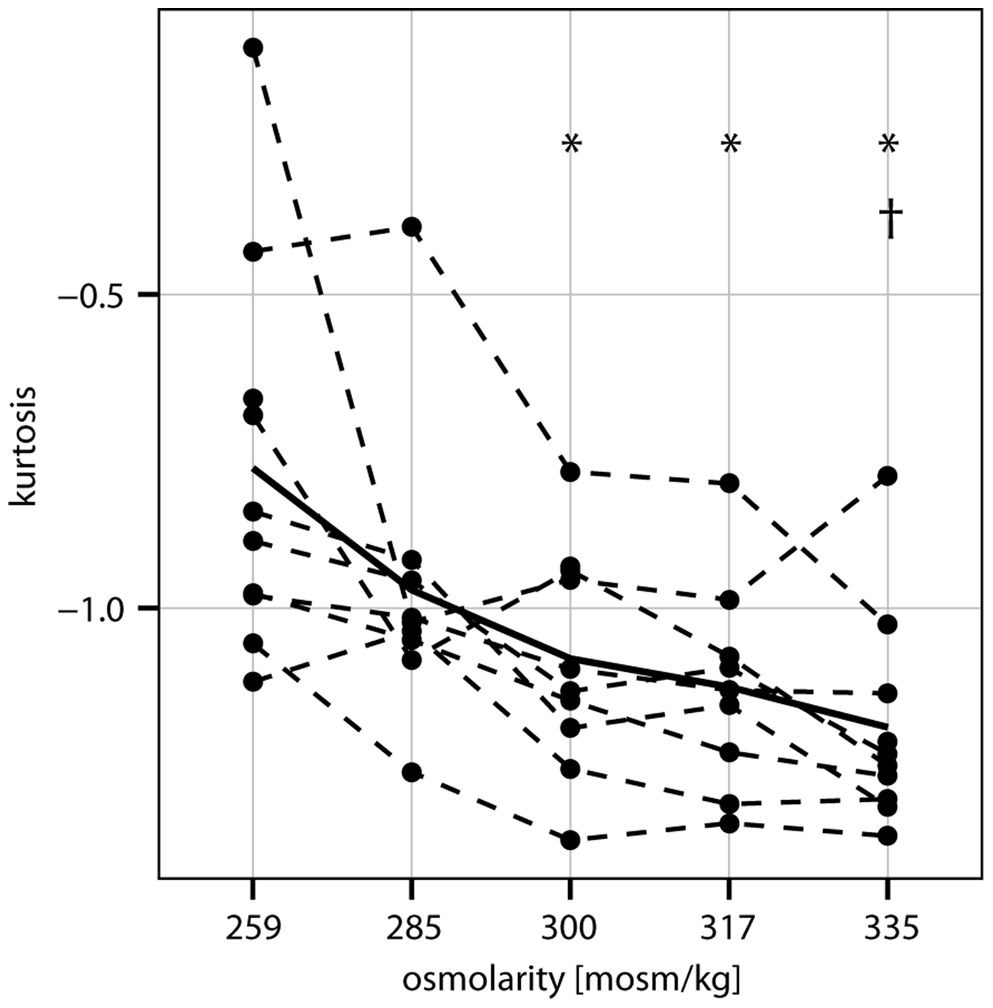
Erythrocyte sphericity and buffer osmolarity. Kurtosis of FSC signal of erythrocytes of 10 subjects of first study part incubated in buffer mediums with varying osmolarity. With increasing osmolarity of buffer medium, sphericity of erythrocytes decreases as indicated by decreasing kurtosis, *indicates significant difference (p<0.05) to kurtosis/sphericity values at 259 mosmol/kg and ^†^indicates significant difference (p<0.05) to kurtosis/sphericity values at 285 mosmol/kg (Friedman-test with adjusted pairwise comparisons).

Impact of buffer osmolarity on erythrocyte shape expressed as kurtosis of FSC signal (mean and individual data) is indicated in Figure 2. Increasing buffer osmolarity and hence decreased sphericity of erythrocytes is displayed by a significant decrease of kurtosis.

For these 50 samples, correlation between kurtosis and Sphi is −0.686 (p<0.05) and correlation between kurtosis and PCD is 0.718 (p<0.05).

### Second Study Part

**Figure 3:**
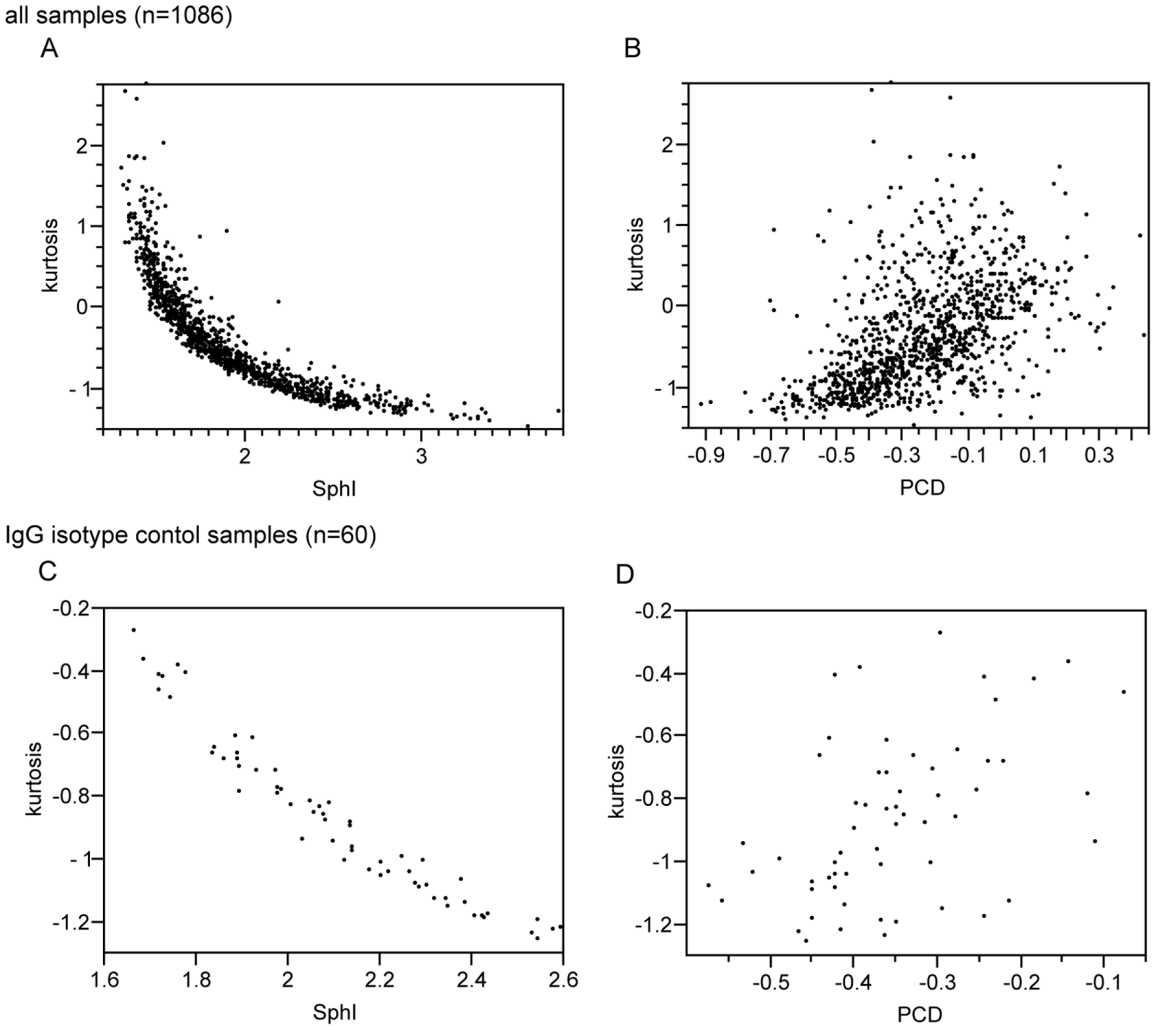
Correlations between respective indicators of sphericity. Scatterplots illustrating the relation between the respective indicators of sphericity in all samples of second study part (A,B) and isotype control samples (C,D). Relation between kurtosis and SphI appears inverse proportional.

**Figure 4:**
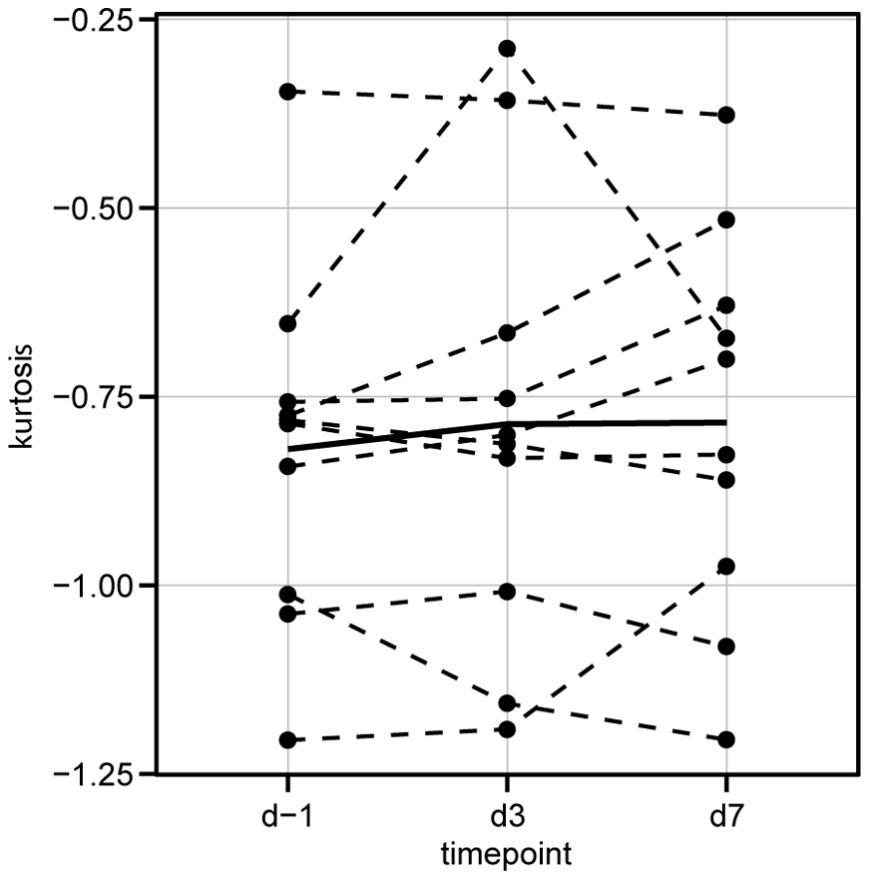
Longitudinal presentation of erythrocyte sphericity. Kurtosis of FSC signal of IgG2b isotype control samples of CONT subjects at all time points. Values of the respective subjects are connected by dashed lines. Solid bold line connects mean values of distribution of kurtosis at the respective time point.

Mean values, standard error of measurement, intra- and inter-individual standard deviation and r ratio for kurtosis, PCD and SphI in isotype control samples IgG2b of CONT are indicated in Table 1. Correlation coefficients between measures of sphericity are indicated in Table 2. Figure 3A, B illustrates correlation between the respective measures in all samples. Figure 3C, D illustrates the correlation between the respective measures in isotype control samples in “Full”. Figure 4 shows the course of erythrocyte sphericity (expressed as kurtosis) in CONT IgG2b-isotype control samples (“Full”) during study period.

**Table 1:** Descriptive statistics for measures of sphericity.

	Mean	SEM	Inter-individualSD	Intra-individualSD	r
SphI	2.19	0.04	0.22	0.12	0.32
PCD	−0.31	0.02	0.12	0.06	0.22
kurtosis	−0.80	0.03	0.23	0.10	0.20

Mean values, SEM, intra- and inter-individual standard deviation (SD) and r ratio in IgG2b isotype control samples (representative for all IgG isotype controls) of CONT.

**Table 2:** Results of correlation analysis between measures of sphericity.

	SphI	PCD	kurtosis
SphI		−0.65* (−0.53*)	−0.97* (−0.98*)
PCD	−0.65* (−0.53*)		0.58* (0.48*)
kurtosis	−0.97* (−0.98*)	0.58* (0.48*)	

Spearman ρ correlation coefficients for the respective sphericity indicators. Values in brackets describe correlation for native erythrocytes (isotype controls).

* Indicates significant correlation (p<0.05).

## Discussion

The key finding of the present study is that kurtosis of FSC histograms of erythrocytes indicates erythrocyte sphericity. Moreover, in a large set of FSC distributions of erythrocytes, kurtosis and SphI are highly correlated. In longitudinal data analysis, both measures feature a similar standard error of measurement. Therefore, it can be concluded that kurtosis of the FSC signal histogram appears as a novel parameter to display sphericity of erythrocytes in flow cytometry analysis.

Our work describes the applicability of sphericity measures in a wide range of FSC distributions - as these measures were applied using an “extreme” population of erythrocytes induced by either variation of buffer osmolarity (first study part) or the density gradient separation, antibody-labelling and variation of flow velocities (second study part).

The pattern of decreased sphericity of erythrocytes caused by increased serum osmolarity has been well described [16]. In the first study part, the feasibility to use kurtosis of FSC histogram as an indicator for erythrocyte sphericity was confirmed. Kurtosis, in parallel to other sphericity indicators, displayed a change of erythrocyte change towards a more spheric form with decreased buffer osmolarity.

The second study part evaluates application of kurtosis against known sphericity indicators in a broad spectrum of FSC distributions: Through density separation, erythrocytes can be separated by their size [17]. Analysis of FSC signal distribution of erythrocytes after density separation shows that different erythrocyte density fractions feature different FSC distributions reflecting the size of erythrocytes [18]. Hence, processing erythrocytes in density gradients leads to a sideward movement of the average of the FSC signal distributions.

In a second step, erythrocytes were incubated with several FITC-labelled antibodies. This step also contributed to achieving various FSC distributions as incubation of erythrocytes with antibodies also leads to relevant alterations of FSC signal distribution [3].

In conclusion, the two-stepped processing of erythrocytes lead to a great variation of the FSC signal as illustrated by the broad distribution range of the measures of sphericity, which clearly exceeds the range of FSC signal obtained through analysis of native erythrocytes as described by Piagnerelli et al. [4], [6]. Through analysis of erythrocytes incubated with FITC isotype controls, which, by definition, do not specifically bind to erythrocyte surface, it was granted that also the normal range of distributions of FSC signals was covered in our study. For the erythrocytes from the FITC-isotype control samples, the range for SphI was similar to the values previously published for healthy subjects (Table 2) [4], [6]. However, PCD values obtained from FITC-isotype samples were remarkably lower than values previously published. Therefore, unspecific binding might have slightly altered the shape of native erythrocytes and our samples might therefore not fully mirror the properties of native erythrocytes.

As kurtosis and SphI are highly correlated in our study, both measures appear to be valid measures of sphericity over a broad range of FSC distributions. In our opinion, use of kurtosis has several advantages over the use of SphI: First, kurtosis is a clearly defined mathematical measure easily applicable in automated data analysis. This is a major advantage over of SphI. In previous work, Piagnerelli and co-workers [4], [6] thoroughly explain calculation of SphI. First, a minimum between the modes of the bimodal distribution must be identified to divide the distribution at that point in two parts. In a second step, the mode of each of the two parts of the distribution is identified and the final cut-off between both parts of the whole distribution is set as the mean of these two modes. SphI is finally calculated as a ratio of the medians of the two parts.

This procedure appears rather complex when compared with the automated calculation of kurtosis which is available in all major statistic software packages at present. Moreover, automated calculation of the mode of a distribution might be challenging and mode is prone to be altered by measurement noise. Comprehensive mathematical solutions to approximate the mode of a distribution are available. Still, all these are approximation formulae and are valid only when the event number is high (as it is usually the case in flow cytometry). Still methodological variability in SphI might arise just from different approximation methods for the mode.

As indicated above, a reference bimodal distribution is needed as a starting point to calculate the two modes of that distribution or analysis. When applying the method on different flow cytometers with the risk of altered channel assignment, or even when comparing cells of different sizes (resulting in a sideward shifting of the FSC distribution as indicated above) this might require a specific calibration procedure. In contrast, kurtosis of a distribution is independent from a sideward shift of the distribution and from a sophisticated gating strategy.

These examples illustrate possible limitations in application of the SphI in automated data analysis. However, it has to be stated that these concerns are solely theoretical: given the high correlation between SphI and kurtosis even in distributions that are far from being bi-or monomodal (where SphI is <2), SphI appears to be a robust predictor of kurtosis.

In their articles, Piagnerelli and co-workers indicate skewness (expressed as PCD) as another indicator for sphericity [4], [6]. Like kurtosis, skewness is a well defined measure [19] which can be calculated automatically. However, Piagnerelli used PCD as an indicator for skewness. Use of skewness for this purpose might have certain limitations. First, application of skewness to describe sphericity implies that the FSC distribution is asymmetrical. For symmetrical distributions, whether bi- or monomodal, skewness is 0. Second, skewness can be automatically calculated, use of PCD is approximating skewness with some error. In our data, where a broad range of FSC distributions were obtained through processing and selection of erythrocytes, PCD is not correlated as highly with SphI (and kurtosis) as in Piagnerelli’s work, especially when SphI is <2 (compare Figure 3.). Taken together, these shortcomings let us prefer kurtosis or SphI as measures of sphericity in comparison to PCD.

Longitudinal data analysis of isotype control samples from “Full” (which were evaluated to control for background fluorescence; these samples were not processed through any density gradient and not incubated with a specific antibody) shows that all measures of distribution have a comparatively low standard error of measurement. Moreover, as a r ratio less than 0.6 in all indicators of sphericity illustrates that the intra-individual variability of sphericity is smaller than the variability of the respective measure in the whole study population [14]. This suggests that erythrocyte sphericity might be a distinct individual characteristic. Therefore, it appears that it might be beneficial to monitor sphericity of erythrocytes longitudinally. Piagnerelli et al. indicated applications for the analysis of erythrocyte sphericity [4]. It might be speculated that e.g. in sepsis monitoring, change of erythrocyte morphology profiles could be used as a longitudinal measure for severity of disease of individual patients. Moreover, longitudinal monitoring of erythrocyte sphericity might contribute to future methods for the detection of autologous blood transfusions in anti-doping, where monitoring of certain biomarkers in individuals over time is already routinely applied [20].

In this context, we emphasize that automated calculation of kurtosis of erythrocyte FSC distribution in haematology analysers appears feasible and might constitute an additional parameter in haematological work-up in the future.

In conclusion we provided evidence that kurtosis of erythrocyte FSC distribution might be a favourable parameter for analysis of erythrocyte sphericity in flow cytometry, mainly because its application appears more practical when compared to SphI and does not have the mathematical shortcomings of skewness in this context.

Moreover we were able to show that intra-individual variability of erythrocyte sphericity is much smaller than inter-individual variability, which makes erythrocyte sphericity a suitable marker for individual longitudinal monitoring.
